# Using skeletal position to estimate human error rates in telemanipulator operators

**DOI:** 10.3389/frobt.2023.1287417

**Published:** 2024-01-09

**Authors:** Thomas Piercy, Guido Herrmann, Angelo Cangelosi, Ioannis Dimitrios Zoulias, Erwin Lopez

**Affiliations:** ^1^ Faculty of Science and Engineering, The University of Manchester, Manchester, United Kingdom; ^2^ Remote Applications in Challenging Environments, United Kingdom Atomic Energy Authority, Culham Science Centre, Oxford, United Kingdom

**Keywords:** bio-mechanical modelling, feedback systems, psychology, sensory integration, applications in industrial activities

## Abstract

In current telerobotics and telemanipulator applications, operators must perform a wide variety of tasks, often with a high risk associated with failure. A system designed to generate data-based behavioural estimations using observed operator features could be used to reduce risks in industrial teleoperation. This paper describes a non-invasive bio-mechanical feature capture method for teleoperators used to trial novel human-error rate estimators which, in future work, are intended to improve operational safety by providing behavioural and postural feedback to the operator. Operator monitoring studies were conducted *in situ* using the MASCOT teleoperation system at UKAEA RACE; the operators were given controlled tasks to complete during observation. Building upon existing works for vehicle-driver intention estimation and robotic surgery operator analysis, we used 3D point-cloud data capture using a commercially available depth camera to estimate an operator’s skeletal pose. A total of 14 operators were observed and recorded for a total of approximately 8 h, each completing a baseline task and a task designed to induce detectable but safe collisions. Skeletal pose was estimated, collision statistics were recorded, and questionnaire-based psychological assessments were made, providing a database of qualitative and quantitative data. We then trialled data-driven analysis by using statistical and machine learning regression techniques (SVR) to estimate collision rates. We further perform and present an input variable sensitivity analysis for our selected features.

## 1 Introduction

In hazardous environments, telemanipulators play a vital role in reducing risks to human operators. Nuclear teleoperations are typically characterised by low automation reliance (Seward and Bakari, 2005; Tokatli et al., 2021) and rely heavily on human-operated systems (Chen and Barnes, 2014). This study focuses on the MASCOT teleoperation system (Skilton et al., 2018) at the UK Atomic Energy Authority’s (UKAEA) Joint European Torus (JET) fusion laboratory.

MASCOT operations require reliable and effective human-telemanipulator interfaces; it is therefore imperative that control complexity and task load parameters are not worsened by any implemented system. Therefore, a non-invasive sensor approach, with an appropriate method of assessing the operator had to be used, where human factors have been considered to be able to test a minimally-invasive monitoring system.

In the development and prototyping process, operator feedback suggested a desire for training tools designed to encourage good habits and any mechanisms that might improve operator health and operational safety. Preliminary sensor data analysis suggests a potential link between operator pose and task load (Piercy et al., 2022), laying the foundation for further investigation.

To explore the relationship between bio-mechanical features and operational safety, a teleoperation task designed to induce operator errors was formulated. This task involved a wire loop buzzer game, similar in design to a surgical robot training task (Liu and Curet, 2015), and a baseline ‘simple’ task. Operator monitoring studies collected bio-mechanical data, task execution times, collision rates, and workload assessments. Machine learning techniques were then applied to estimate operator safety factors based on bio-mechanical features, offering the potential for real-time safety warnings in shared control models.

Szczurek et al. (2023) state that the success of teleoperations hinges on three key aspects: Operators must effectively achieve mission goals, adapt to unexpected challenges while minimising risks to human life and equipment, and remain aware of their changing capabilities throughout the task. They further assert that these factors are closely tied to the operator’s emotional and physiological state, which in turn impacts the operational success rate. In line with this and other works (Chen and Barnes, 2014), we included a task load measurement to analyse any confounding effect of subjective task load on operator bio-mechanical data.

The aims of this research are to investigate the relationships between bio-mechanical features and task performance, to investigate and present relationships for more targeted further study, and to improve operator monitoring systems based on the previous points. This publication makes significant contributions in two key areas. Firstly, it advances bio-mechanical feature analysis for observing operators during telerobotic tasks. A novel collision-rate estimation system is introduced, monitoring operator bio-mechanical pose and estimating collision rates based on non-intrusive sensors. Secondly, it offers a comparison of operators through various metrics, shedding light on task performance and factors influencing operator proficiency. The experiments were conducted in a demanding environment at the JET facility in RACE, providing valuable insights for improving human-telemanipulator interaction in a real setting.

Section 2 describes the telemanipulator, sensor setup, experimentation, and data processing. Section 3 presents results, while section 4 and section 5 offer discussions and conclusions, respectively.

## 2 Materials and methods

### 2.1 System description

**FIGURE 1:**
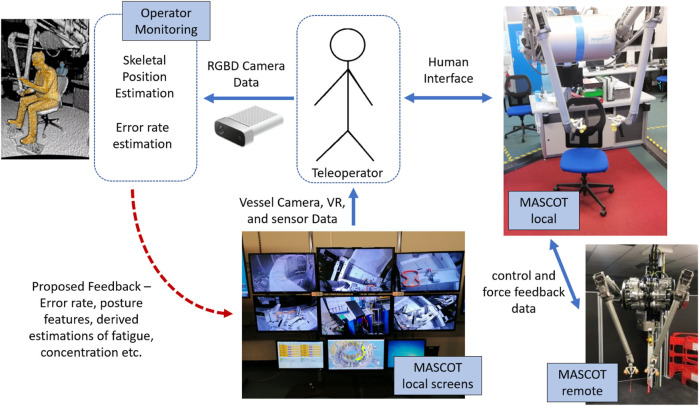
System overview presenting main components of the human-telemanipulator interface along with the experimented operator monitoring system and the proposed entry point for operator feedback. The robot is controlled and exchanges data with the operator via the MASCOT local. The local telemanipulator exactly matches the remote’s state and mirrors inputs from the operator. The operator monitoring block using the depth camera is our contribution. The red dotted arrow represents planned further work.

The system has five main components that interact: the telemanipulator (MASCOT remote side), the local manipulator (the MASCOT local side), the visual interface, the operator, and the operator monitoring described in this paper. See Figure 1 for further detail.

MASCOT (MAnipolatore Servo COntrollato Transistorizzato) (Skilton et al., 2018) is a bilateral manipulator robot where the local side is in a control room and the remote side is mounted on a retractable boom arm that reaches inside the reaction vessel of the JET fusion reactor (see Supplementary Figure S1). It is operated by one primary operator who physically manipulates the arms of the local system (see Supplementary Figure S2) and two secondary operators. The remote MASCOT exactly mirrors the movements of the local MASCOT.

Cameras are used to see inside the reaction vessel and the MASCOT is also tracked via a digital twin that is displayed to the operators. The secondary operators are responsible for monitoring, boom arm and general system control, and camera aiming.

Azure Kinect depth-camera devices were used as a non-invasive sensor to record the participants as they completed the tasks. The pointcloud recordings were used to estimate skeletal position of each participant (Kar, 2011) (see Supplementary Figure S3) - this device is validated for accurate skeletal tracking in bio-mechanical studies (Clark et al., 2019).

### 2.2 Experimental data gathering

The studies were conducted *in situ* using the MASCOT teleoperation system at UKAEA RACE. A total of 14 operators were recorded using RGBD cameras for a total of approximately 8 h, each completing two tasks. Operators were also asked to complete questionnaires. During the wire loop task, collisions were recorded and timestamped. A repeated measures design (all participants do all tasks) was used, where the monitored variables were skeletal position, as estimated from depth camera data, questionnaire responses, and time and number of errors made. The Kinect devices were placed on the same reference marker for each operator—2.3 m to the front and 2.0 m to the right of the MASCOT base—and were mounted on a tripod making it 1.63 m from the ground. The two tasks consisted of dexterity tests to be completed using the MASCOT telemanipulation device; this took less than 40 min per participant. A total of 14 participants were sampled from the available operator staff from RACE, but only 13 results were fully useable. Written informed consent was obtained from all participants in line with the School of Engineering Research Ethics Committee, University of Manchester, who also approved the study.

Participant information was gathered at the start of the experiment. This asked for teleoperation experience in hours, dominant hand, height in metres, and hours of experience. After each task, the participants were asked to complete a NASA-TLX questionnaire (Hart and Staveland, 1988) and the Stanford sleepiness scale (Shahid et al., 2012).

Both tasks were designed to mimic standard training operations. The first task was the wire-wrapping task: This asked the participant to wrap a wire around pegs in a specified pattern. The second task was a wire loop buzzer game, which is similar to a training task used for a surgical telemanipulation device (Liu and Curet, 2015), but was a novel challenge for the MASCOT operators. The objective of this game was to guide wire loops along a wire course without allowing them to touch.

The wire-wrapping task counted how many pegs the participant could wrap a wire around in a specified pattern in a given time limit of 10 min. Clear instructions were given to participants with regard to the pattern and the parameters for testing.

The wire loop game timed how long it took each participant to guide a wire loop along a wire path. Collisions between the wire loop and the wire path caused an LED to light. The times of the collisions were recorded as they happened by time-stamping them to the recording of the operator. This experiment took between 15 and 25 min for each participant. The participants were given instructions on scoring and timing.

### 2.3 Data study

The purpose of this data study is to investigate links between estimated skeletal position and operators’ errors. The data study presented here is, therefore, a trial analysis, and it is suggested that methodologies may be improved upon in further work.

The data gathering study produced a dataset (3.4 TB) of 30 Hz depth camera recordings to be processed. The first step of this was skeletal position estimation using code based on the Microsoft Azure Kinect development kit (Microsoft, 2023). This resulted in 8 h of skeletal tracking data. A computer-assisted observational analysis was first used to identify any occlusion, loss of tracking, and drift. Any such errors were noted and the times that the errors occurred were excluded from the data. A double exponential smoothing filter (Tang and Wang, 2016) was then applied to all readings.

The proposed features for examination, based on previous work (Abdel-Malek and Yu, 2001; Lowes et al., 2013; Solomon and Wang, 2015; Liu et al., 2017; Pulgarin et al., 2018) and qualitative analysis, are operator posture and arm movement dynamics. By extracting specific motion parameters, we aim to gain insights into the kinematic patterns exhibited during the tasks as they relate to the rate of collisions. Data-driven techniques have proven successful in HRI and driver-monitoring tasks (Pulgarin et al., 2018; Wang et al., 2018) and also more specifically in human action recognition using skeletal position estimation (Barkoky and Charkari, 2022).

Therefore, a number of data-driven methodologies including SVM, k-means classification, and incorporating time-series data using distance-based approaches were trialled. Ultimately, we could not reliably classify error events with these methods. We next trialled regression analysis methods which seek to match a function to an output based on dataset features. In this case, we took the collision rate to be the time-series output and trialled a kernel-based regressive machine-learning solution (Regressive Support Vector Machine) for collision rate estimation.

Support Vector Machines (SVMs) (Cristianini and Ricci, 2008) operate in a high-dimensional space and function by maximising the separation margin between classes or the distance to the nearest training data points. In the context of skeletal data analysis, SVMs offer an effective approach for bounding different features extracted from the skeletal position data (Pulgarin et al., 2018). SVR (Regressive SVM) (Yu and Kim, 2012; Zhang and O’Donnell, 2020) models operate by maximising margins from input vectors to a function in order to define an appropriate hyperplane to describe the desired output while fitting the error margin to an acceptable threshold (*ϵ*). In this way, SVR aims to optimally minimise the distance from an estimated function output to the true output—in this case the collision rate by optimising the weight and bias coefficients of the input feature vectors. A radial-based kernel function was used to accommodate both a non-linear output response and a highly dimensional cross-sectional input (Aizerman, 1964; Francis and Raimond, 2021). As we are using time-series data, it is highly likely that the current estimation will be similar to the previous estimation. For this reason, we included some properties of the previous 5 s of estimation as a feature vector.

The hyper-parameters of SVR are *ϵ*, *C*, and *γ*. The Parameter *ϵ* controls the width of the insensitive tube around the prediction hyperplane (i.e., allowable noise in training), *C* controls the number of support vectors selected, and *γ* controls the kernel size. We used the Keras tuner O’Malley et al. (2019) to select optimal hyper-parameters through a random search algorithm.

#### 2.3.1 Data labelling

We recorded three states for each operator: resting, operating, and error made. Resting states were recorded when operators locked the telemanipulator into place and released the input controls. Resting states were excluded from data processing. The collision label started 2 s before a collision was made and lasted for 5 s. The collision rate was then a derived value equal to the mean of the previous 30 s of collision data—it was calculated as a moving average over 900 frames (30 s at 30 Hz) for each operator:

$$Average\;collision\;rate=\bar{R_{c}}=\frac{\sum_{i=n-900+1}^{n}P_{i}}{900},$$

where 900 is the number of data entries over 30 s, P is the value of collision at each data point—either zero or one, and n is the total number of entries. The average collision rate was around one collision per 10 s (0.123 Hz), so 30 s was selected as a time window to minimise regions of the collision rate output with a value of zero.

#### 2.3.2 Feature selection and extraction

Features can be derived from various aspects of the skeletal estimation, such as joint angles, velocity profiles, or joint positions. These features then serve as the input to the SVR and are the independent variables for regression, allowing for meaningful collision rate estimation. A time-series feature allows the model to take previous estimations as an input. Spinal posture (lordosis and vertical alignment) and arm movement dynamics were selected for feature extraction, along with an input vector derived from previous estimations in the time series.

Spinal lordosis (curve) and vertical alignment (lean) are identified as key parameters in optimal sitting biomechanics for vehicle drivers (Harrison et al., 1999) and work requiring concentration and focus (Bhatnager et al., 1985; Drury et al., 2008), which suggests validity for task performance estimation. Arm movement dynamics are identified as a key parameter for a number of studies tracking arm fatigue (Takahashi et al., 2006; Hincapié-Ramos et al., 2014) and general arm movement characterisation (Vermeulen et al., 2002; Clark et al., 2019), including in teleoperation studies (Li et al., 2018), which also suggests validity for task performance estimation.

Spinal parameters were derived by isolating the spine skeletal position estimates: neck, chest, navel, and pelvis, see Supplementary Figure S3. These label points were analysed trigonometrically to derive spinal lean and curve. Lean (Spinal vertical alignment) was calculated as a measure of the total angle of deflection from upright (determined by Azure Kinect accelerometer) between the pelvis and neck. This is a measure of how upright the operator is sitting:

$$\begin{array}{ll} & \;Z-axis\;displacement\;from\;pelvis\;to\;neck\\ & =z_{\mathrm{neck}}-z_{\mathrm{pelvis}}=z_{\mathrm{disp}}, \end{array}$$

$$Planar\;displacement\;from\;pelvis=p=\sqrt{\left(x_{\mathrm{disp}}^{2}+y_{\mathrm{disp}}^{2}\right)},$$

$$Lean=L=tan^{-1}\left(\frac{p}{z_{\mathrm{disp}}}\right).$$

See Supplementary Figure S4.

The spinal curve (lordosis) was derived by measuring the cumulative total angle of deflection between the pelvis and neck—it is the sum of the magnitude of the angle of deflection from each joint to the next (from the pelvis to the neck). This is a measure of how ‘hunched’ the operator is. The deflection is calculated by:

$$\begin{array}{ll} & \;Height\;of\;Pelvis\;to\;Navel\;link\\ & \;=\Delta z_{\mathrm{pelvis-navel}}=z_{\mathrm{Navel}}-z_{\mathrm{Pelvis}}, \end{array}$$

$$\begin{array}{ll} & \;Pelvis\;to\;Navel\;planar\;displacement\\ & =p_{\mathrm{pelvis-navel}}=\sqrt{{\left(x_{\mathrm{navel}}-x_{\mathrm{pelvis}}\right)}^{2}+{\left(y_{\mathrm{navel}}-y_{\mathrm{pelvis}}\right)}^{2}}, \end{array}$$

$$Deflection=d_{\mathrm{pelvis-navel}}=tan^{-1}\left(\frac{p_{\mathrm{pelvis-navel}}}{\Delta z_{\mathrm{pelvis-navel}}}\right).$$

This deflection is calculated for each link: Pelvis to the navel, navel to chest, and chest to neck. The total curve is then the sum of these deflections,

$$Total\;curve=C_{\mathrm{total}}=\overset{\mathrm{neck}}{\underset{\mathrm{pelvis}}{\sum }}deflection.$$

See Supplementary Figure S5.

This process was repeated for both task datasets for each participant, giving a baseline spine lean and curve, as well as a spine lean and curve, over a time series during the error-inducement task. When one is subtracted from the other, we are left with a relative deviation from the baseline for each participant; the purpose of this step is to normalise the data across participants to allow for comparison over the time series.

The arm movement dynamics were extracted by isolating the arm skeletal position and rotation estimates: neck, shoulder, elbow, wrist, and hand. Each of these is given as a relative displacement from the previous joint. Velocity, acceleration, angular velocity, and angular acceleration were calculated for each point through numerical differentiation with regard to the previous sample period (at 30 Hz). No steps were taken to normalise these values across participants as they describe overall motion dynamics, which is what is being examined.

The time series features are three values derived from the SVR outputs. These values are the previous discrete estimation:

$${\hat{R}}_{\left(n-1\right)},$$

the mean of the previous 5 s of estimations:

$$\frac{\sum_{i=n-150}^{n-1}{\hat{R}}_{i}}{150},$$

and the value of the previous estimation subtracted from the estimation 5 s ago:

$${\hat{R}}_{\left(n-150\right)}-{\hat{R}}_{\left(n-1\right)}.$$

#### 2.3.3 Collision rate estimation models

**FIGURE 2:**
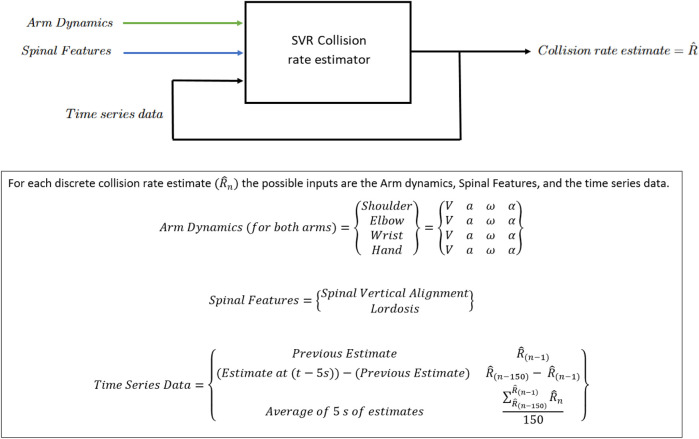
SVR model system diagram.

The collision rate is to be estimated for every next sample at 30 Hz $\left(Sampling\;period=\tau =0.0\dot{3}\;s\right)$ , using the previously discussed SVR over the time series. The discrete output of the SVR $\left({\hat{R}}_{n}\right)$ is a periodical estimation of the 30-s average error rate, where the training objective is the average rate of collisions over the previous 30 s. See Figure 2 for an input and output overview of the SVR model.

Five tests were done to evaluate model effectiveness. One test was completed to establish a baseline, and three further tests were conducted as a preliminary independent variable sensitivity analysis. These tests are designed to address our primary aim of investigating the relationships between bio-mechanical features and task performance. As part of a secondary aim inspired by Pulgarin et al. (2018), one further test was conducted to evaluate the model’s suitability for extrapolation. The results of the testing are Mean Absolute Error (MAE) and Root Mean Square Error (RMSE) which are used to compare model fitness (Chai and Draxler, 2014).

**FIGURE 3:**
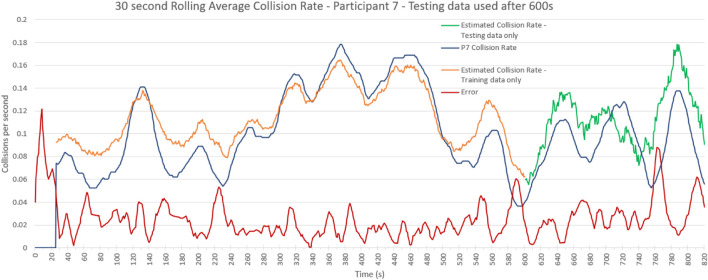
Collision Rate Estimation Example. Participant 7–true collision rate and estimated collision rate showing separated testing data in green. This model is trained with all feature data on the first 70% of the data and collision rate is estimated for the remaining 30%. The RMSE for the testing data is 0.026.

The baseline test uses data from each participant individually and estimates the collision rate of that participant. For training, 70% of the data was used, and validation was done with the remaining 30%. Input vectors were arm dynamics features, spinal features, and time-series features. This test was run on each participant and used to evaluate a collision rate estimation model for an individual. An example model output is given in Figure 3 of the results.

Sensitivity tests were conducted to evaluate the model’s sensitivity to the selected independent variables. All sensitivity tests were conducted using 70% of the data for training, and validation was done with the remaining 30% and was conducted for each participant. The sensitivity tests are similar to the baseline test but omit a feature vector.

The first sensitivity test does not use any time-series knowledge. Input vectors are spine features, and arm dynamics features only. The second sensitivity test does not use any spinal features, and the third sensitivity test does not use any arm dynamics features. The purpose of these tests is to evaluate the effect of the missing input on the model.

The extrapolation test was conducted to evaluate the generalisability of the collision rate estimator by estimating the collision rate of a participant using a group-generated model: The test uses data from all participants and estimates the collision rate of one unknown participant. All input vectors were used, and this test was run on each participant.

## 3 Results

### 3.1 Testing results

Supplementary Table S1 shows the overview of the testing results of each participant–participant 11 is excluded from analysis. The maximum number of collisions during the wire loop game task was 149, the minimum was 76, and the average was 102 with a standard deviation of 19.6. The average time taken was 832 s (13.9 min) with a standard deviation of 210 s (3.5 min); the minimum time taken was 597 s (10 min), and the maximum was 1443 s (24 min).

### 3.2 NASA TLX responses

Supplementary Table S2 shows NASA-TLX results which examine the self-reported workload, and these results are presented graphically in Supplementary Figure S6. In the NASA TLX assessment methodology, the ratings range from 0% to 100%. Groups were also separated by experience, with the requirements being more than 1000 h of experience, less than 1000 h but more than 100, and less than 100 h of experience. Separated groups are represented graphically in Supplementary Figure S7.

Beginners reported higher mental, physical, and temporal demands compared to experts. Both beginners and experts often reported higher felt performance than intermediate operators. Experts also reported significantly lower effort compared to others, while frustration levels were relatively consistent across all groups.

### 3.3 Bio-metrics

There was no detectable relationship between groups separated by handedness, height, or sleepiness. Raw data are shown in Supplementary Table S3. Sleepiness was rated using the Stanford sleepiness scale—modified to use the same marking scheme as NASA-TLX. A total of 11 out of 13 participants were right-handed (P11 excluded), average height was 1.77 m, and average sleepiness was 2.9 on a modified Stanford sleepiness scale. This indicates ‘Functioning at high levels but not at the peak and ability to concentrate’ (see Supplementary Table S4).

### 3.4 Collision rate estimation

#### 3.4.1 Sensitivity analysis

The collision rate estimator results in Table 1 show the testing results for individual interpolation along with tests where an independent variable was excluded. Presented is the mean number of collisions per second, the mean absolute error (MAE), and the RMSE (Root Mean Square Error) in collisions per second for each participant under each testing condition.

**TABLE 1:** Error Rate Estimation Sensitivity Test results table: Showing SVR results from testing data (30% of each data set) with feature sets removed. Presenting the average number of collisions per second and the Mean Absolute Error (MAE) and RMSE (Root Mean Square Error) in collisions per second. The mean standard deviation and range of these values are also presented.

		All data included (baseline)		Time-series data excluded		Spine data excluded		Time-series data excluded	
	Mean collision rate	MAE	RSME	MAE	RMSE	MAE	RMSE	MAE	RMSE
p1	0.124	0.023	0.030	0.122	0.133	0.016	0.020	0.040	0.044
p2	0.090	0.022	0.027	0.085	0.097	0.021	0.026	0.026	0.034
p3	0.153	0.033	0.041	0.141	0.155	0.024	0.031	0.043	0.056
p4	0.132	0.025	0.032	0.124	0.135	0.019	0.023	0.036	0.042
p5	0.117	0.026	0.032	0.112	0.124	0.022	0.026	0.038	0.048
p6	0.146	0.033	0.040	0.136	0.151	0.024	0.032	0.052	0.069
p7	0.103	0.021	0.026	0.098	0.107	0.019	0.022	0.029	0.035
p8	0.103	0.027	0.033	0.099	0.111	0.023	0.028	0.040	0.049
p9	0.084	0.019	0.023	0.081	0.088	0.019	0.023	0.027	0.035
p10	0.130	0.024	0.031	0.121	0.132	0.018	0.023	0.038	0.049
p12	0.155	0.030	0.037	0.148	0.156	0.020	0.025	0.036	0.048
p13	0.148	0.025	0.031	0.138	0.147	0.019	0.023	0.035	0.047
p14	0.112	0.021	0.026	0.109	0.117	0.017	0.021	0.033	0.044
Mean	0.123	0.025	0.031	0.116	0.127	0.020	0.025	0.036	0.046
Std. Dev	0.023	0.004	0.005	0.021	0.021	0.002	0.004	0.007	0.009
Range	0.071	0.014	0.018	0.068	0.068	0.008	0.013	0.026	0.035

The lowest average RMSE and MAE were obtained where Spine data was not used. The RMSE and MAE range and standard deviation were also the lowest for this group. The next lowest RMSE and MAE were using all data, then arm dynamics excluded, and then the time-series data excluded group.

Presented in Figure 3 is an example of the error rate estimator against the true error rate for one participant. This estimation model is trained on the first 70% of the data and collision rate is estimated for the last 30% using all feature data. The RMSE for the testing data is 0.026 collisions per second.

#### 3.4.2 Extrapolation

The collision rate estimator (Supplementary Table S5) shows the testing results for the entire group against an unknown individual.

### 3.5 Other notes

Participant 11 was excluded from all processing as they did not follow instructions during the wire loop task. Participant feedback indicated that the wire loop task-induced concentration, frustration, fatigue, and a sense of competition. The wire wrapping task was simple and did not induce such responses, making it potentially suitable as a baseline. There were also observable trends such as the number of errors appearing to negatively correlate with the time taken, indicating that slower operators committed fewer errors. During the debriefings, participant feedback seemed to indicate that posture and work position may have been influencing performance and fatigue felt and that fatigue may have been influencing posture, equally.

## 4 Discussion

NASA-TLX results indicate some methodological validity as the wire loop task was able to produce a varied response across experience ranges, and it was found to be similarly frustrating by all participants. This suggests that task difficulty, not experienced task load, was the primary collision-rate influence. It is important to note that these results are self-reported and should be interpreted cautiously, considering factors like over or under-confidence (McKendrick and Cherry, 2018).

Additional questionnaires provided data for normalisation but have not shown significant correlations with specific features. Further investigation is needed, particularly considering individual differences such as height, handedness, and sleepiness, which could impact body dynamics and lead to a more generalised model.

Using the regression models, we were able to demonstrate that there is a detectable and consistent property of arm dynamics that can be used to predict the error rate for this task for individual operators. The regression model proved most effective when using participants’ own data to estimate their collision rates. The generalisation tests did not demonstrate error rate prediction results significantly better than random (Mean RMSE = 0.073). Individual differences in error reactions may contribute to this inaccuracy, suggesting the need for new techniques or features to enhance model generalisation.

RMSE values from the feature sensitivity testing allow for a comparison between models. The mean RMSE values, given in the number of collisions per second, across participants, were 0.031 (all data), 0.127 (no time-series data), 0.025 (No spine data), and 0.046 (no arm dynamics data). From this, we can infer that arm dynamics and time-series data played significantly more influential roles than posture on model accuracy.

Due to the use of the kernel trick (Aizerman, 1964; Francis and Raimond, 2021), it is not possible to directly examine the relationships between the estimation and specific feature variables (Üstün et al., 2007). In this instance, it was a necessity to use a kernel-based methodology as the data was both cross-sectional and in a time series, and the required output was a complex polynomial. Since there does seem to be enough vector data to moderately accurately estimate the collision rate, it would suggest that further study is warranted.

Comparison of RMSE between participants indicates that more accurate models can be made for some participants than others; this may imply that the behaviour of some participants is more consistent than others. The accuracy of the results gained through support vector machines for body motion analysis is comparable to other works in this domain (Perego et al., 2009; Alghowinem et al., 2013; Shetty and Rao, 2016), which implies that the information gained by the skeletal estimation is suitably discriminating for individual operator performance estimation.

As all on-site testing was completed within 1 h for each participant, it is possible that the success of the estimation model is due to very consistent input data. This means that the regression model may be over-suited for that particular task on that particular day and factors such as clothes, day of the week, tiredness, fluctuations in mood, and health may all have a significant impact on the model accuracy in a multi-day scenario.

The findings from these results could have a significant impact on industrial telemanipulation safety though it is only a first step. A generalisable and all-purpose human error detector for manipulation would be a very desirable tool though much more development would be needed in a number of fields for this to become feasible. The results presented here show that using a non-invasive sensing solution, it is possible to build a collision rate estimator for an individual for one task. Currently, the potential uses for such an estimator are for the purposes of a monitored, repeated training exercise and for monitoring very repetitive tasks. With further studies, it may be possible to construct more generalisable estimators in two domains: generalisability to people and generalisability to tasks. If it is possible to generalise across people, this tool could be used as a feedback method as part of a training program or be used for operator performance evaluation. If it is possible to generalise tasks, then this tool could be trained for an individual and deployed for improved operational safety, as well as health and safety monitoring.

## 5 Conclusion

This paper contributes to the methodology for human-telemanipulator interface evaluation, incorporating the bio-mechanical pose, the NASA TLX assessment method, questionnaires, task execution time, and recorded collisions. It introduces novel approaches for identifying operator errors and evaluating operator performance. The insights gained here can guide research in other human-telemanipulator interaction applications and contribute to the field of AI estimation for enhanced operational safety in high-risk environments. Future work will explore different analysis techniques, including Neural-Network and Deep Learning approaches, with data from additional field studies.

Due to the non-invasive bio-mechanical feature capture methodology, the techniques explored here can easily be applied to many other domains such as medical teleoperations. Further studies could also explore the integration of other data sources such as physiological sensors, Infrared sensors, or eye tracking cameras.

The telerobotic tasks in hazardous and sensitive environments are a source of increased operator stress and workload. Therefore, the design, development, and evaluation of human-centred robot interfaces play an essential role in the success of reliable and safe operations. This publication has presented important first steps aimed at improving the monitoring process for telemanipulator operators and informing the subsequent developments of human-telemanipulator interfaces.

## Data Availability

The datasets presented in this article are not readily available because of a UKAEA and project funding agreement. Data available upon request to author after 01/01/2025. Requests to access the datasets should be directed to TP thomas.piercy@manchester.ac.uk.
