# Supplementary material for: Using skeletal position to estimate human error rates in telemanipulator operators
Source: Front Robot AI. 2024 Jan 9;10:1287417. doi: 10.3389/frobt.2023.1287417 (PMC10803571; doi:10.3389/frobt.2023.1287417)
Supplement: Supplementary file 1 [file DataSheet1.PDF]

Supplementary Material

1 SUPPLEMENTARY TABLES AND FIGURES

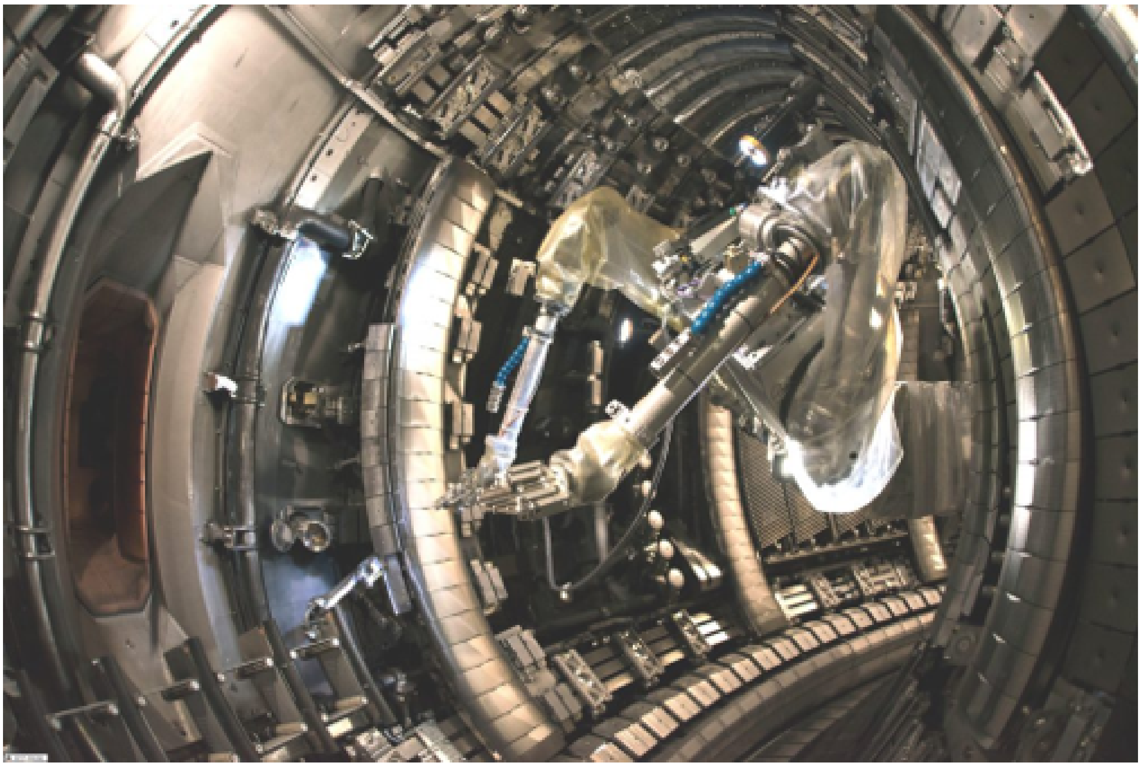

Figure S1. Remote side of MASCOT showing in-vessel operation

P#	Hand	Height (m)	Experience (h)	Notes	Wrap_Score	Collisions	Time (s)
**1**	left	1.71	60	Estimated	23	88	673
**2**	right	1.83	45	Known	29	132	1443
**3**	right	1.58	20	Known	22	149	838
**4**	right	1.81	20	Known	13	101	759
**5**	right	1.81	120	Estimated	15	95	815
**6**	right	1.85	100	Estimated	17	105	675
**7**	right	1.54	40	Known	22	82	816
**8**	right	1.7	40	Estimated	26	101	972
**9**	right	1.7	7500	Estimated*	29	76	896
**10**	right	1.78	1000	Estimated	34	81	597
**11****	right	1.78	3000	Estimated	31	47	110
**12**	left	1.85	26	Estimated	15	99	624
**13**	right	1.94	100	Estimated	24	111	749
**14**	right	1.7	37.5	Known	20	107	958

Table S1. Testing results overview. Notes indicates if experience is known or estimated, Wrap score shows how many pegs the wire was wrapped through in the wrapping task, Collisions and time both refer to the wire loop task. *between 5 000 and 10 000 hours estimated. **Participant did not follow testing instructions, and is excluded from analysis

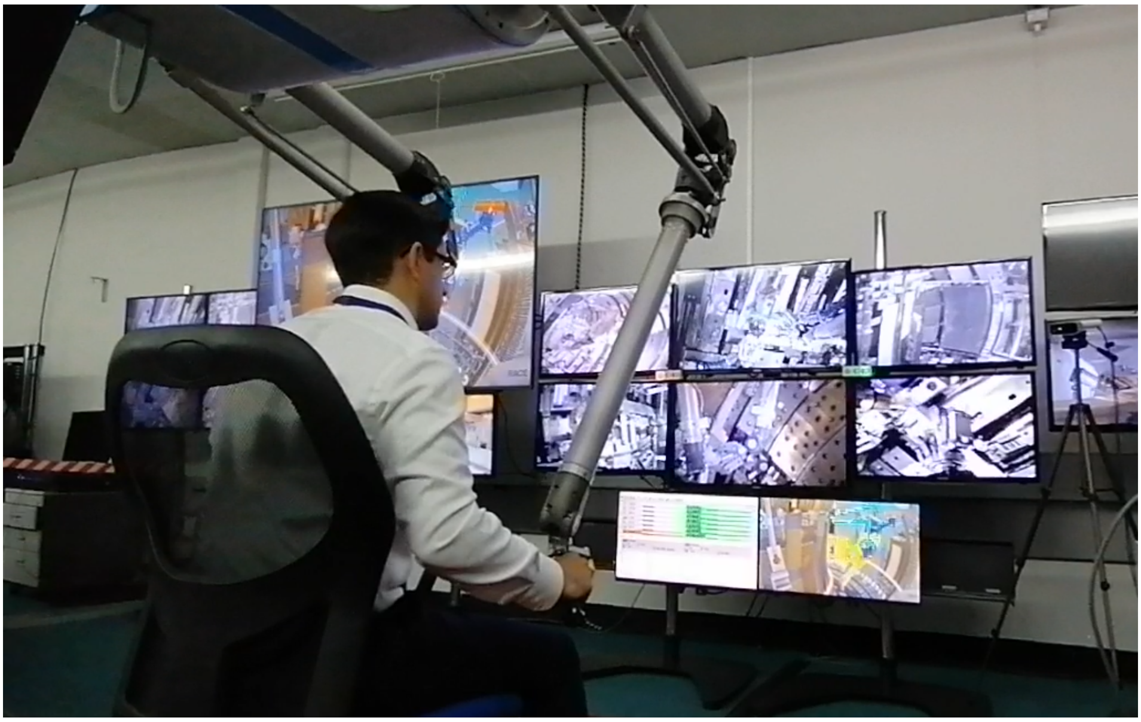

Figure S2. Local side of MASCOT showing human physical and visual interface in the JET control room

P#	Mental Demand	Physical Demand	Temporal Demand	Performance	Effort	Frustration
**1**	73%	33%	67%	42%	68%	68%
**2**	93%	98%	77%	57%	98%	67%
**3**	87%	58%	67%	82%	87%	92%
**4**	73%	92%	52%	62%	73%	33%
**5**	88%	63%	42%	24%	81%	68%
**6**	61%	42%	49%	12%	77%	86%
**7**	84%	70%	59%	40%	89%	54%
**8**	87%	72%	62%	73%	87%	57%
**9**	67%	35%	23%	83%	27%	42%
**10**	49%	49%	49%	76%	27%	50%
**12**	62%	77%	33%	57%	67%	22%
**13**	57%	77%	28%	77%	78%	38%
**14**	75%	75%	60%	64%	80%	70%

Table S2. NASA-TLX results, task load responses for each participant

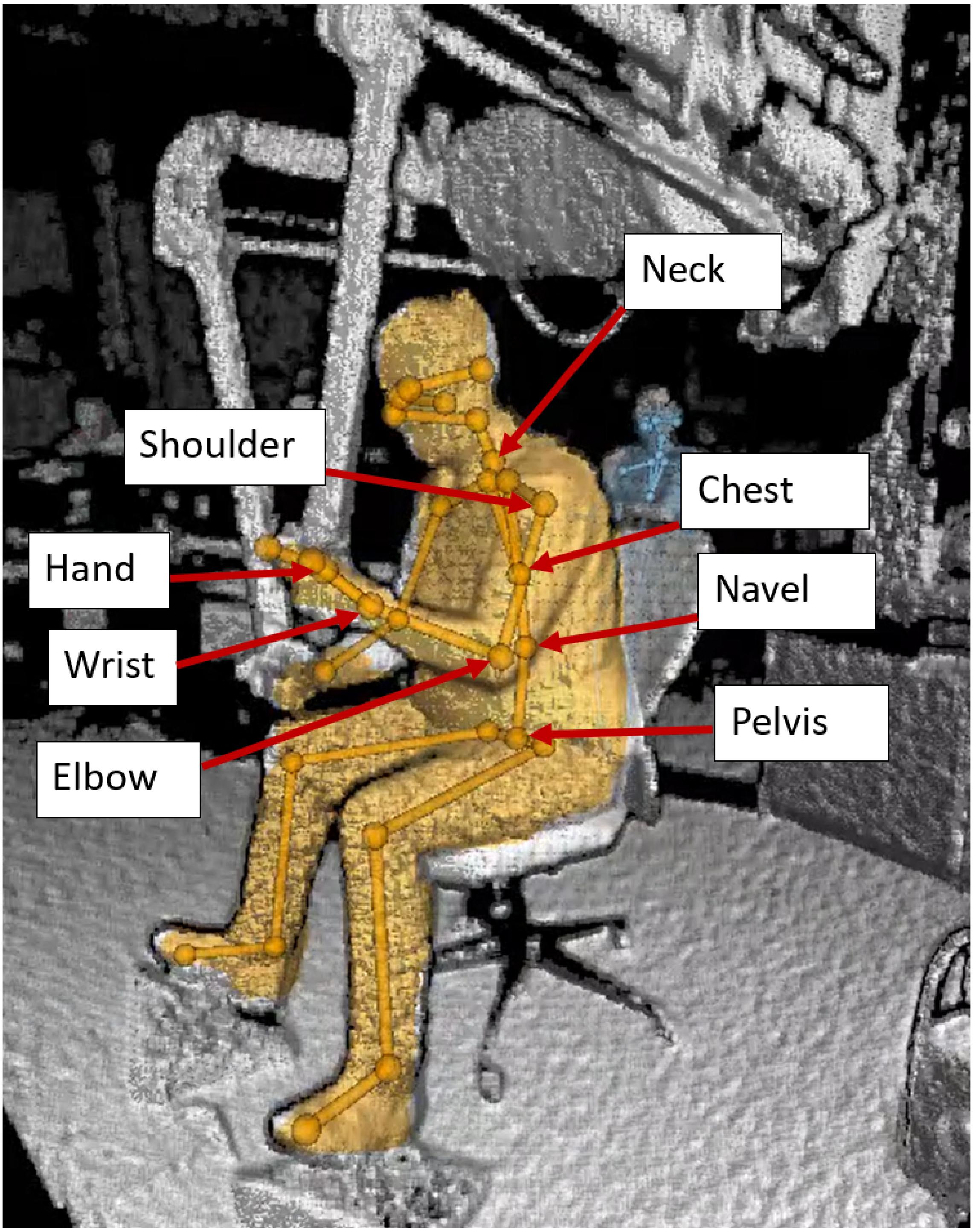

Figure S3. MASCOT operator constructed depth image with skeleton position estimation overlay. Key joints are labelled.

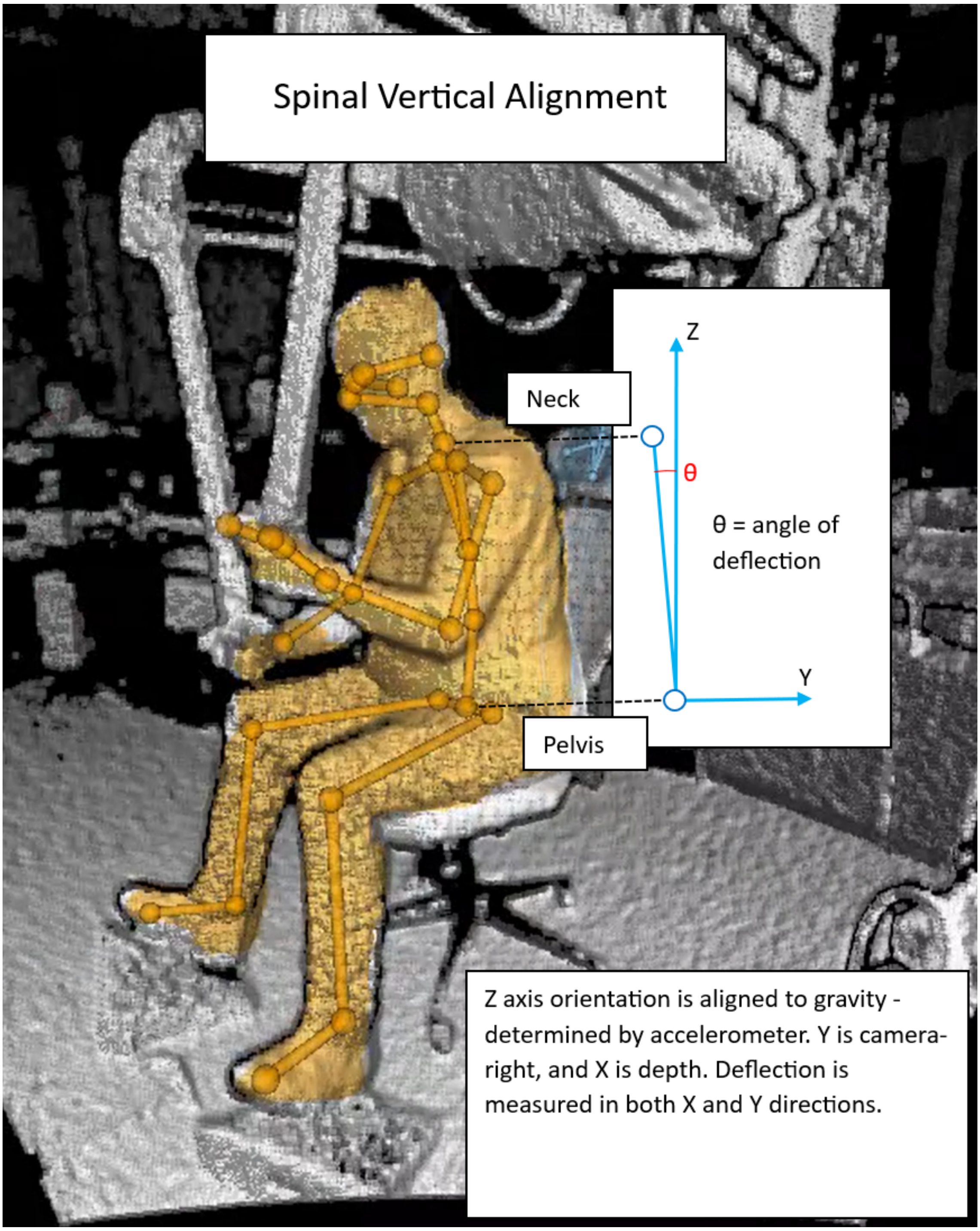

Figure S4. MASCOT operator constructed depth image with skeleton position estimation overlay. Spinal vertical alignment derivation is shown.

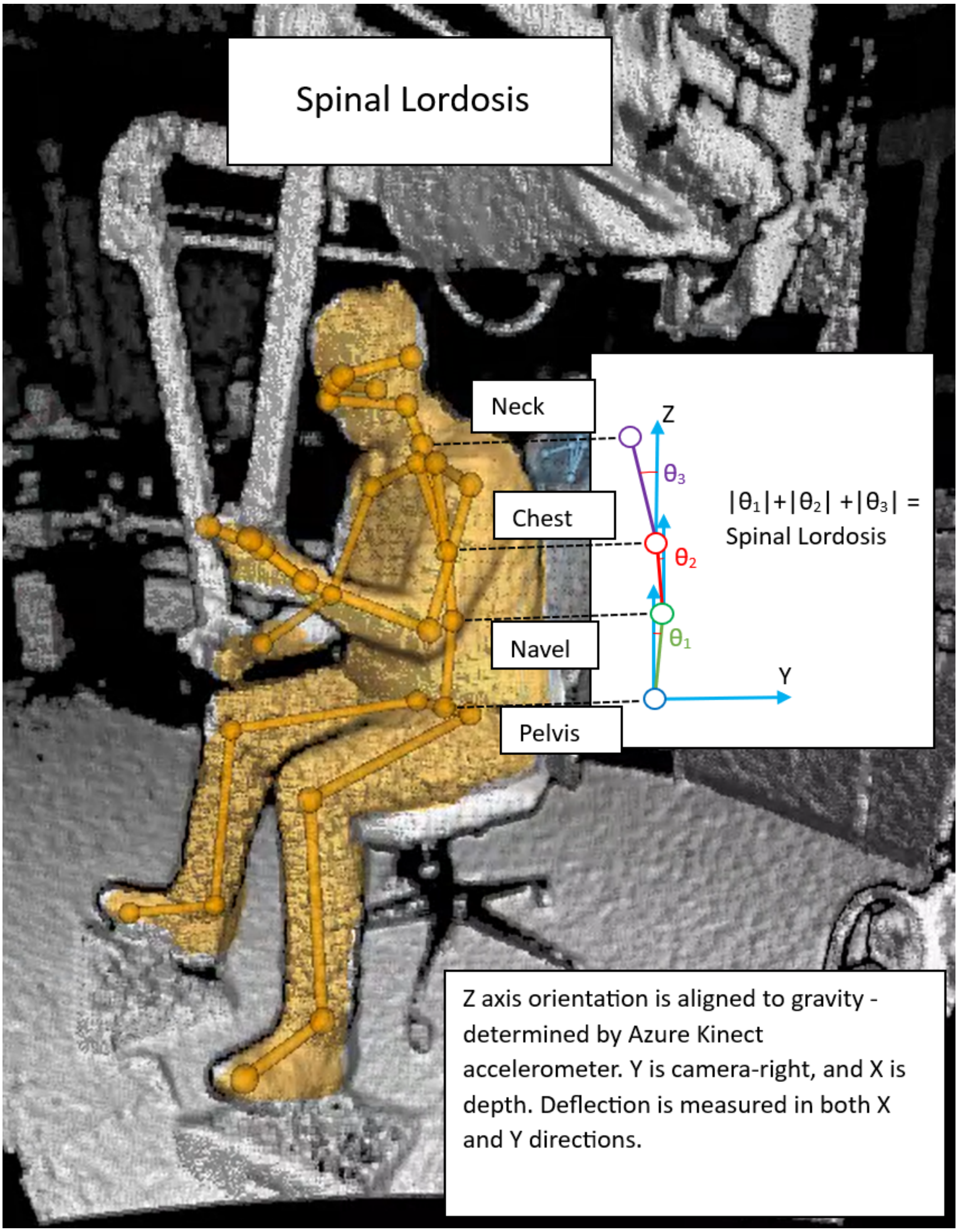

Figure S5. MASCOT operator constructed depth image with skeleton position estimation overlay. Spinal lordosis derivation is shown.

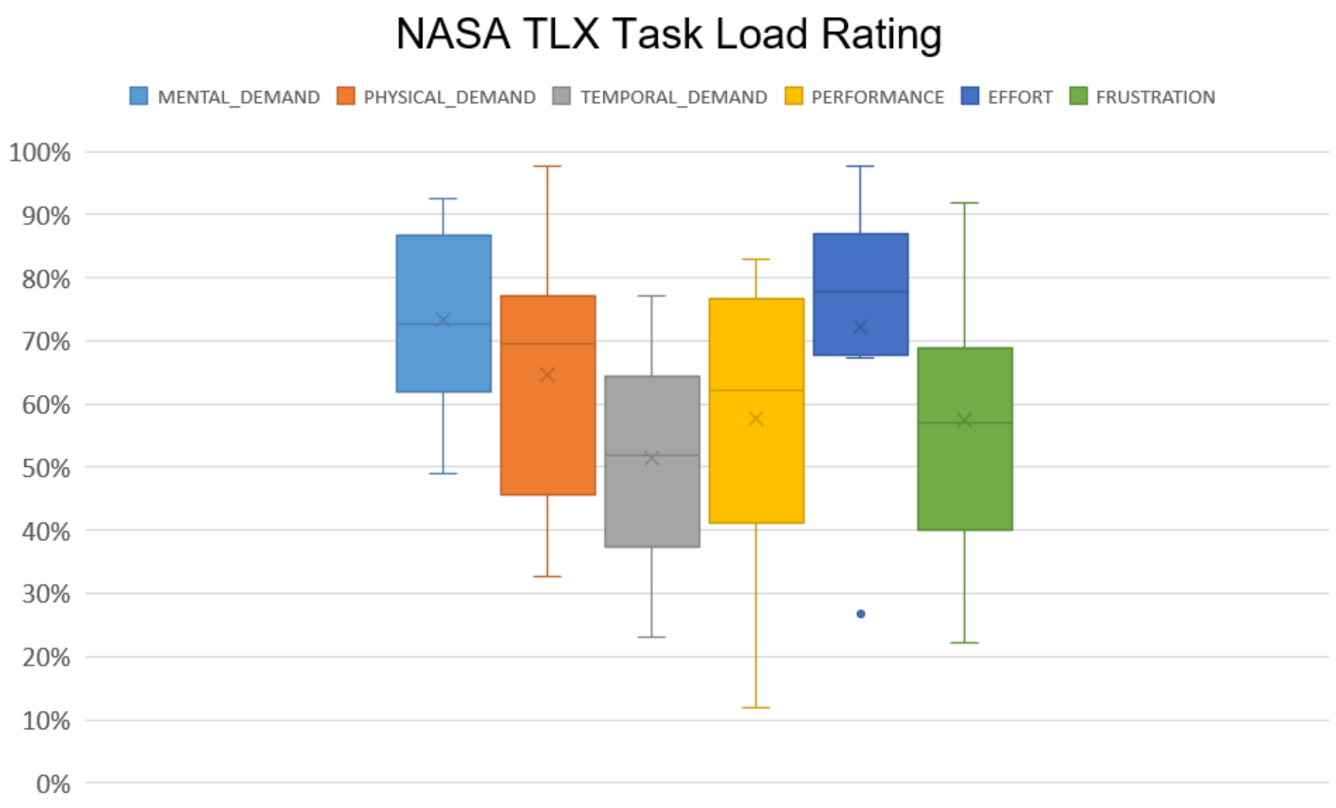

Figure S6. NASA TLX results overview for all participants. The scale for ratings was 0-100%

Participant no.	Handedness	Height (m)	Sleepiness
P1	l	1.71	1.7
P2	r	1.83	3.9
P3	r	1.58	3.6
P4	r	1.81	3.5
P5	r	1.81	2.4
P6	r	1.85	1.6
P7	r	1.54	2.8
P8	r	1.70	3.5
P9	r	1.70	3.5
P10	r	1.78	3.8
P11	r	1.78	2.8
P12	l	1.85	2.2
P13	r	1.94	2.9
P14	r	1.7	2.8

Table S3. Table showing recorded participant biometrics - Handedness, height(m), and sleepiness - as estimated by a modified version of the Stanford Sleepiness scale

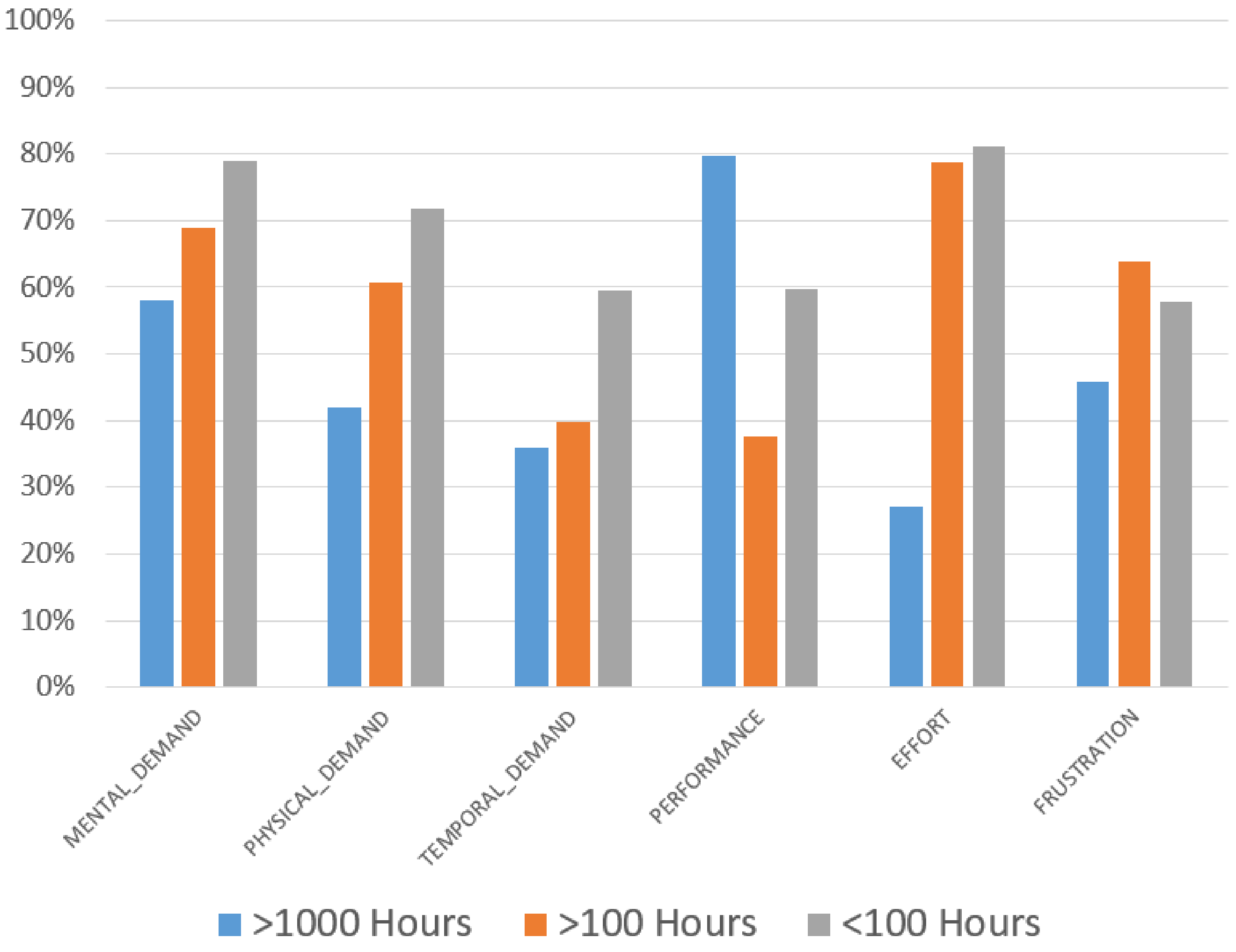

Figure S7. NASA TLX results showing task load rating split into groups by hours of experience

Description	Response Range
Feeling unusually alert, hyperactive, stimulated	0 - 1.1
Feeling active, vital, alert, or wide awake	1.1 - 2.2
Functioning at high levels, but not at peak; able to concentrate	2.2 - 3.3
Awake, but relaxed; responsive, but not fully alert	3.3 - 4.4
Somewhat foggy, let down	4.4 - 5.5
Foggy; losing interest in remaining awake; slowed down	5.5 - 6.6
Sleepy, woozy, fighting sleep; prefer to lie down	6.6 - 7.7
No longer fighting sleep, sleep onset soon; having dream-like thoughts	7.7 - 8.8
Asleep	8.8 - 10

Table S4. Table showing how to interpret Stanford sleepiness scale results

P#	Mean collision rate	MAE	RMSE
**p1**	0.124	0.069	0.071
**p2**	0.090	0.070	0.073
**p3**	0.153	0.091	0.095
**p4**	0.132	0.073	0.080
**p5**	0.117	0.058	0.059
**p6**	0.146	0.064	0.098
**p7**	0.103	0.059	0.062
**p8**	0.103	0.050	0.058
**p9**	0.084	0.044	0.046
**p10**	0.130	0.053	0.055
**p12**	0.155	0.095	0.118
**p13**	0.148	0.058	0.068
**p14**	0.112	0.064	0.065
**Mean**	0.123	0.065	0.073
**Std Dev**	0.023	0.014	0.019
**Range**	0.071	0.051	0.072

Table S5. Error Rate Estimation Extrapolation Test results table: Showing SVR results from testing data (100% of each participant). Presenting average number of collisions per second, and the MAE (Mean Absolute Error) and the RMSE (Root Mean Square Error) in collisions per second.
